# Development of a PK/PD–Efficacy Modeling Framework for Covalent Inhibitors

**DOI:** 10.3390/ph19081228

**Published:** 2026-08-04

**Authors:** Nashid Farhan, Indranil Rao, Jan Wahlstrom, Upendra P. Dahal

**Affiliations:** 1 Pharmacokinetics & Drug Metabolism and Bioanalytical Sciences, Amgen Inc., South San Francisco, CA 94080, USA; 2 Pharmacokinetics & Drug Metabolism and Bioanalytical Sciences, Amgen Inc., Thousand Oaks, CA 91320, USA

**Keywords:** covalent inhibitors, PK/PD, mechanistic modeling, KRAS G12C, EGFR, sotorasib

## Abstract

**Background/Objectives:** Covalent inhibitors often demonstrate prolonged pharmacological effects even after their disappearance from the site of action because target recovery depends on target turnover. This disconnect between pharmacokinetics (PK) and pharmacodynamics (PD) complicates the development of such inhibitors since plasma exposure coverage of in vitro potency cannot be used for compound selection and human dose projections. In this study, we describe the development of a PK/PD modeling framework for covalent inhibitors. **Methods:** The model was developed for KRAS G12C inhibitors using pre-clinical data on sotorasib. The model was validated using data from both internal Amgen compounds and published data for several KRAS G12C inhibitors. The applicability of the framework was extended to EGFR covalent inhibitors by incorporating PK/PD and the efficacy of osimertinib and its active metabolite AZ5104. **Results:** The model successfully captured the pharmacokinetics, KRAS G12C target occupancy, inhibition of phosphorylation of ERK protein, and tumor growth inhibition following the administration of sotorasib in mice bearing MIA PaCa-2 xenografts. External validation with several internal Amgen compounds as well as publicly available data on KRAS G12C inhibitors showed the robustness of the model. The application of this framework to EGFR inhibitor Osimertinib and AZ5104 captured p-EGFR dynamics and resultant tumor growth inhibition. **Conclusions:** A modeling framework for covalent inhibitors was developed that links exposure to target occupancy, downstream signaling, and tumor efficacy. This framework could be useful for compound optimization and human dose projections.

## 1. Introduction

**Figure 1:**
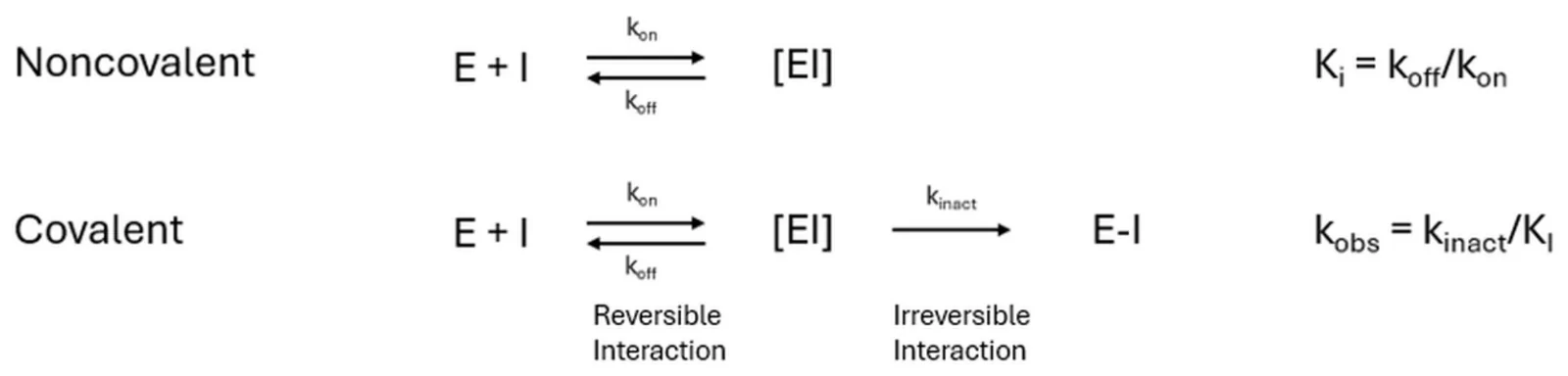
Binding mechanism for noncovalent and covalent drugs. E is enzyme, I is inhibitor, k_on_ is binding rate, k_off_ is dissociation rate, K_i_ in inhibition constant for reversible inhibitors, K_I_ is the inhibition constant for covalent inhibitors, and k_inact_ is rate of inactivation.

Small molecule drugs are typically classified as either reversible (noncovalent) or irreversible (covalent) inhibitors based on their binding mechanisms. Reversible inhibitors associate with enzymes via non-covalent interactions such as hydrogen bonding, electrostatic attractions, and van der Waals forces, which result in the transient formation of an enzyme–inhibitor complex. The potency of reversible inhibition is quantified by the inhibition equilibrium constant (K_i_), as shown in Figure 1. In contrast, covalent inhibitors initially form an enzyme–inhibitor complex through similar interactions; this process is defined by the equilibrium constant K_I_, followed by an additional step that establishes an irreversible covalent bond with the target protein, the formation of which is characterized by a rate constant, k_inact_, as illustrated in Figure 1. The first step of covalent interaction is the non-covalent binding of a drug with the target protein, while the second step is the formation of a covalent bond between enzyme and drug to form enzyme–inhibitor adduct, which is irreversible, and the rate of such a reaction is monitored as inactivation rate, k_inact_. It is worthy to note that the covalent drugs can be subdivided into reversible and irreversible covalent agents. For reversible covalent drugs, the covalent bond formed is temporary and subject to dissociation, whereas for irreversible covalent drugs, the bond is permanent, resulting in sustained protein inactivation. Figure 1 only depicts the irreversible covalent drugs, which is the scope of this manuscript. The restoration of protein function after irreversible inhibition requires the de novo synthesis of the protein. Covalent drugs have the potential to offer several notable advantages over noncovalent alternatives, including sustained pharmacological activity, increased target selectivity, and the effective engagement of difficult binding sites, resulting in growing interest in covalent modalities within pharmaceutical research. This manuscript discusses irreversible covalent inhibitors. For clarity, the term “covalent inhibitors” will refer to irreversible covalent inhibitors in the following sections.

Covalent drugs have been in use since the late 19th century, although their mechanisms of action remained largely unidentified until more recent decades. Aspirin serves as an early example of a covalent inhibitor, with its acyl transfer mechanism for cyclooxygenase inactivation elucidated in the mid-1970s [1]. The development of targeted covalent inhibitors (TCIs), which are purposefully designed to interact with specific protein targets, began in the early 2000s [2]. This advancement was marked by the approval of ibrutinib—targeting Bruton’s tyrosine kinase (BTK)—and afatinib—targeting the epidermal growth factor receptor (EGFR)—by the US Food and Drug Administration in 2013 [3].

Covalent drugs are often distinguished by a notable disconnect between pharmacokinetic (PK) and pharmacodynamic (PD) properties. For reversible drugs, the PD outcome is contingent upon the concentration of drug available to dynamically and reversibly bind to the target protein. Once the drug is depleted at the site of action, and if there is no delay between PK and PD, no further PD effect is anticipated as the protein regains its functionality in the absence of the inhibitory agent. In contrast, covalent drugs can exert PD effects long after their disappearance from the site of action owing to irreversible covalent binding with the target protein, which leads to the sustained inactivation of the protein’s function. Hence, the continuous presence of the covalent drug at the site of action becomes unnecessary once the target protein has been inactivated, and the restoration of catalytic function requires the resynthesis of the protein. Consequently, the duration of the PD effect for covalent drugs lasts until the target protein is fully resynthesized, making the protein resynthesis rate a key determinant of PD duration and dosing frequency; longer protein half-lives permit less frequent dosing intervals. In summary, the PD effect of a covalent drug depends not only on exposure (drug concentration), but also on the inactivation parameters (such as inhibition constant K_I_, inactivation rate k_inact_), protein synthesis rate, protein concentration, protein half-life, and other system-specific factors. Therefore, PK/PD modeling for covalent drugs is a particularly complex process.

PK/PD modeling supports structure–activity–relationship (SAR)-driven compound optimization and predicts human doses associated with efficacy, serving as a fundamental component of drug discovery projects. The PK of a drug is influenced by its absorption, distribution, metabolism and excretion (ADME) characteristics, while the PD effects are determined by drug concentration, binding kinetics and system parameters at the target site. The PK/PD models can be constructed to forecast the time course of PD effects based on drug dosing and exposure. Given the complexity of covalent drug actions, multiple in vitro and in vivo parameters should be considered in PK/PD modeling. Quantitative mechanistic PK/PD models have been used to predict the PK/PD profiles of covalent drugs [4,5,6], though existing published models typically address individual drugs, and their broader application remains unknown. This work describes the development of a mechanistic PK/PD model for covalent drugs, validated with several compounds targeting one protein, and further applied to other target proteins to assess the generalizability of the model.

## 2. Results

### 2.1. Data Source—Brief Description of In Vitro and In Vivo Studies—Sotorasib

The half-life of the KRAS G12C protein was reported to be approximately 20–24 h, and a value of 22 h was adopted in the model. The concentration of KRAS G12C protein in MIA PACA-2 tumor cells was determined to be 1.65 ± 0.32 nM by LC-MS/MS assay. The half-life of p-ERK was very short: approximately 5 min based on the in vitro inhibition of p-ERK in the presence of different upstream inhibitors. The maximal rate of inactivation (k_inact_) and equilibrium dissociation constant for sotorasib were reported to be 3060 h^−1^ and 86 µM, respectively [7]. For some of the other inhibitors, such experimental data were not available. Instead, the potency of those compounds was evaluated in terms of k_obs_/[I] (k_obs_: apparent first-order rate of inhibition). Since K_I_ values for these compounds were generally around 100 µM, which was much higher than C_max_, we assumed that k_obs_/[I] = k_inact_/K_I_. Therefore, if only k_obs_/[I] were available for any Amgen compounds, we assumed k_inact_ = k_obs_ ×100 uM and K_I_ = 100 µM for simulation purposes.

The in vivo data source for PK/PD model development was taken from a previous publication by Canon et al. [7]. Unbound concentrations for both plasma and tumor samples were obtained by multiplying their respective values by f_up_ (fraction unbound in plasma) and f_ut_ (fraction unbound in tumor). Tumor-to-plasma K_p,uu_ values were calculated as the ratio of unbound tumor to unbound plasma exposure, using either concentrations or the corresponding unbound area under the curves.

### 2.2. Model Development

**Figure 2:**
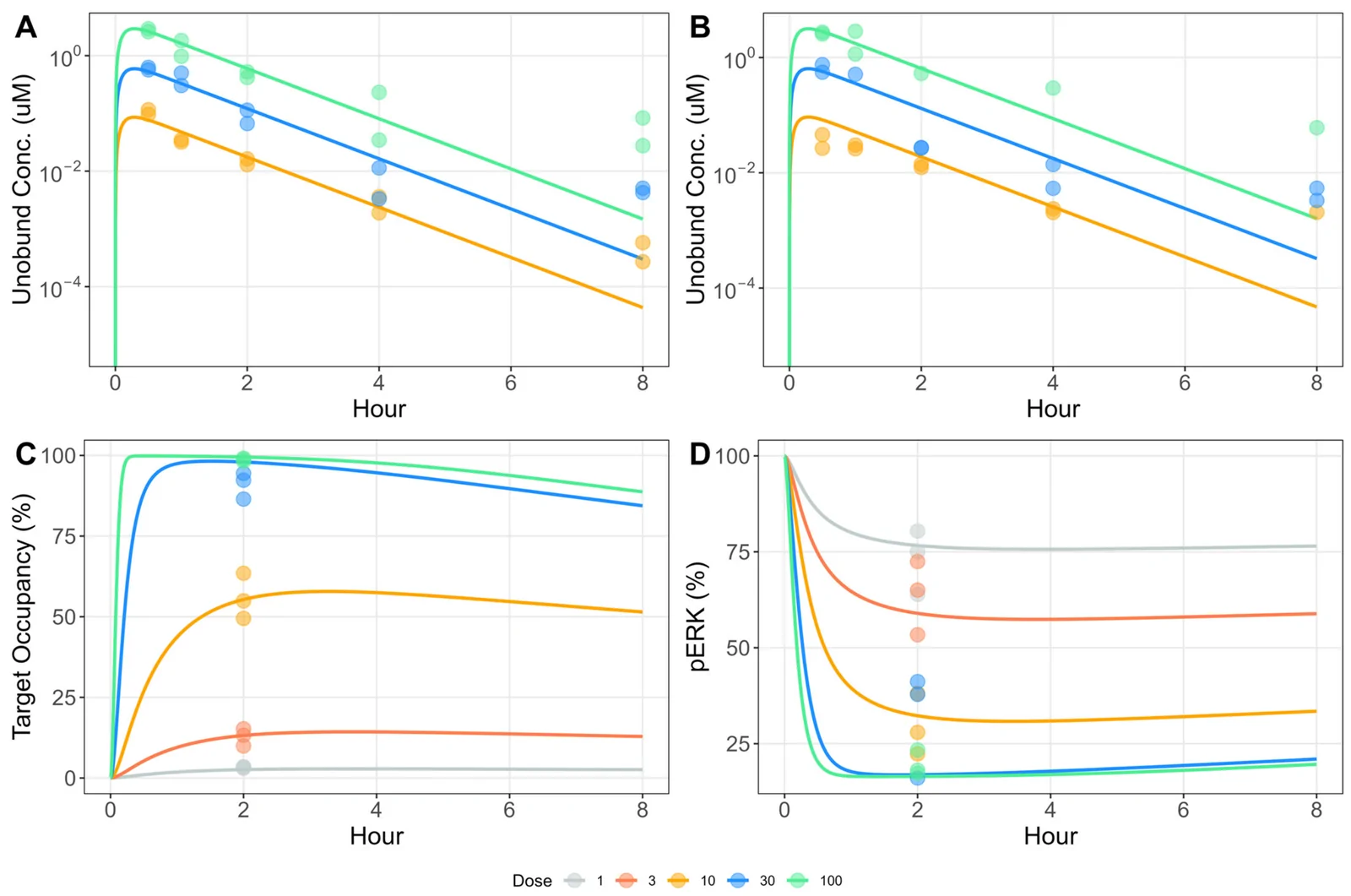
Unbound plasma concentrations (**A**), unbound tumor concentrations (**B**), target occupancy (**C**), and phospho-ERK inhibition (**D**) following administration of sotorasib in Mia PaCa-2 xenograft bearing mice. Solid lines are model predictions and the solid circles are observations.

The pharmacokinetics of sotorasib were described by a one-compartmental model with first-order absorption. Even though the observed PK demonstrates a bi-phasic profile, a one-compartment model was selected because the two-compartment model resulted in non-identifiable parameter estimates. The absorption rate constant (k_a_) could not be determined due to the lack of observed plasma concentrations during the absorption phase. Therefore, k_a_ was assumed to be 1. The unbound CL and V_c_ were estimated to be 5.41 L h^−1^kg^−1^ and 45.82 L kg^−1^, respectively (Table 1). The one-compartment model underpredicted plasma concentrations at the 8 h time point. However, this underprediction is unlikely to significantly affect the PD parameter estimates because the PD data were collected at 2 h. The model successfully captured plasma concentrations at earlier time points where PD data (%TO and p-ERK) were available, allowing the development of PK–PD correlation (Figure 2). The transfer of drugs from the plasma compartment to the xenograft tumor compartment was assumed to be instantaneous (since no delay in C_max_ between plasma- and tumor-concentration profiles was observed for sotorasib and other Amgen KRAS G12C covalent inhibitors) (Figure 2A,B). Therefore, unbound plasma-tumor clearance, kT, was fixed to 1000 L h^−1^ in the model (the corresponding equations are provided in the Supplementary Materials). This large, fixed value was used as a numerical approximation to impose rapid plasma–tumor equilibration and should not be interpreted as a physiologically meaningful clearance parameter. The tumor volume was fixed to the xenograft tumor volume used in the PK/PD study: approximately 200 mm^3^. The other parameter needed to describe unbound tumor concentration was the ratio between unbound tumor to unbound plasma concentrations (K_P,uu_). A single K_P,uu_ value for all three dose levels was estimated simultaneously with Vc_u_ and CL_u_. The estimated K_P,uu_ was 1.08, meaning no significant retention of sotorasib in the tumor.

Subsequently, the PK model parameters were fixed, and k_on_ was estimated as 10.56 µmole^−1^ h^−1^. Based on this estimate, koff was calculated as 908.16 h^−1^. The model was able to capture the observed %TO of sotorasib with a slight overprediction for 30 mg kg^−1^ dosing level (Figure 2C). Next, the p-ERK indirect response model was developed, in which %TO of drug to KRAS G12C protein modulated the phosphorylation of ERK. The hill coefficient of this model (Equation (5)) was estimated to be 0.34, and the model was able to capture the observed p-ERK inhibition in a mouse model (Figure 2D).

**Figure 3:**
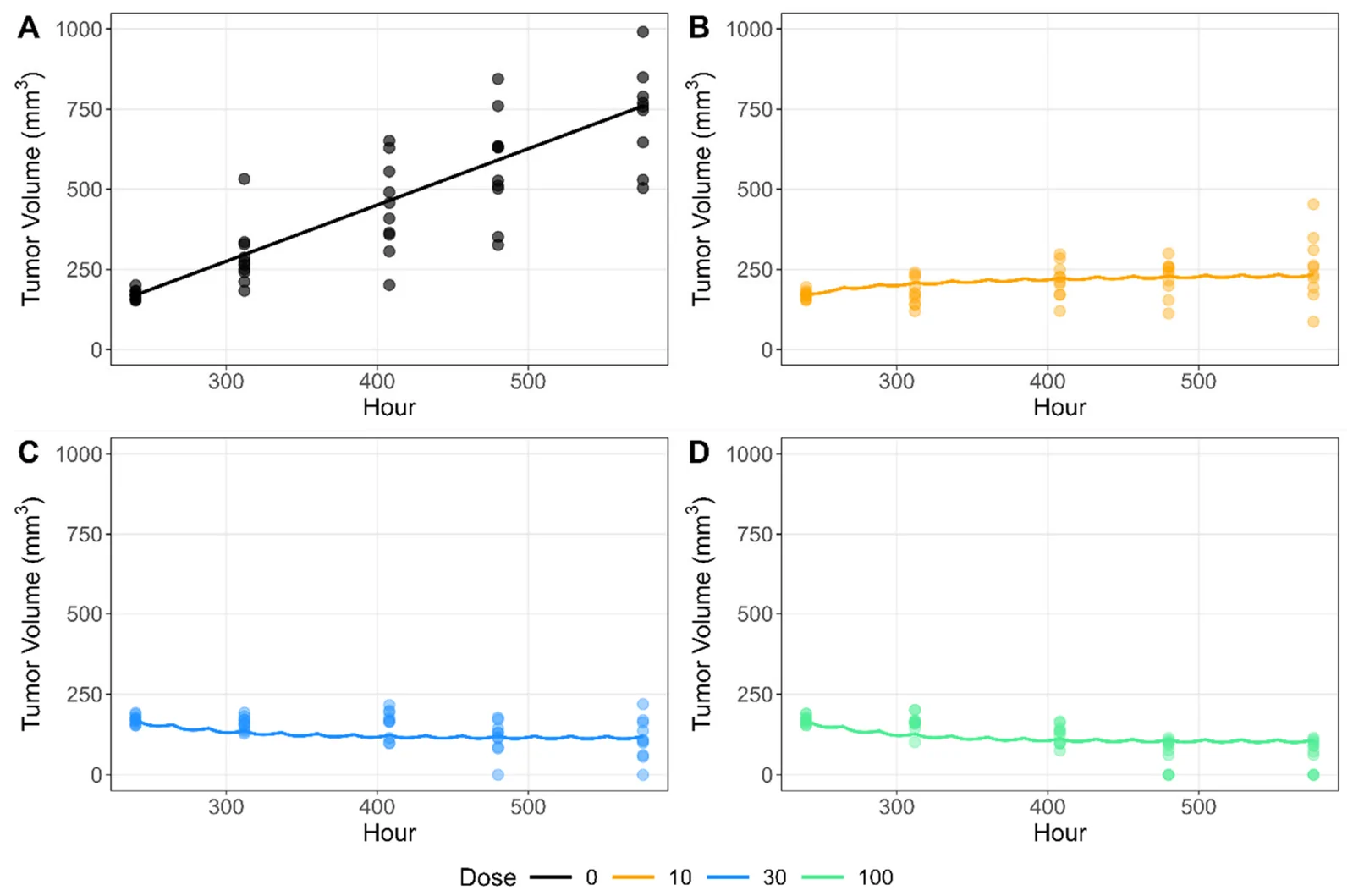
Efficacy of sotorasib following repeated daily oral dosing of 0 mg/kg (**A**), 10 mg/kg (**B**), 30 mg/kg (**C**), and 100 mg/kg (**D**) in Mia PaCa-2 xenograft-bearing mice. Solid lines are model predictions, and the solid circles are observations.

A zero-order tumor-growth model was selected because tumor growth was approximately linear over the observed volume range in the placebo groups from both the sotorasib and internal Amgen compound efficacy studies. The zero-order growth rate (kg) was estimated as 1.75 mm^3^ h^−1^ utilizing the observed xenograft MIA PaCa-2 tumor growth following placebo administration. Tumor growth inhibition was described by a first-order inhibition model driven by the fraction of p-ERK inhibition (Equation (6)). The model successfully captured sotorasib-induced tumor growth inhibition at all dose levels (Figure 3). The first-order tumor growth inhibition rate (k_kill_) and log of hill coefficient in this model were estimated as 0.088 h^−1^ and 0.81, respectively (Table 1).

### 2.3. Model Validations

**Figure 4:**
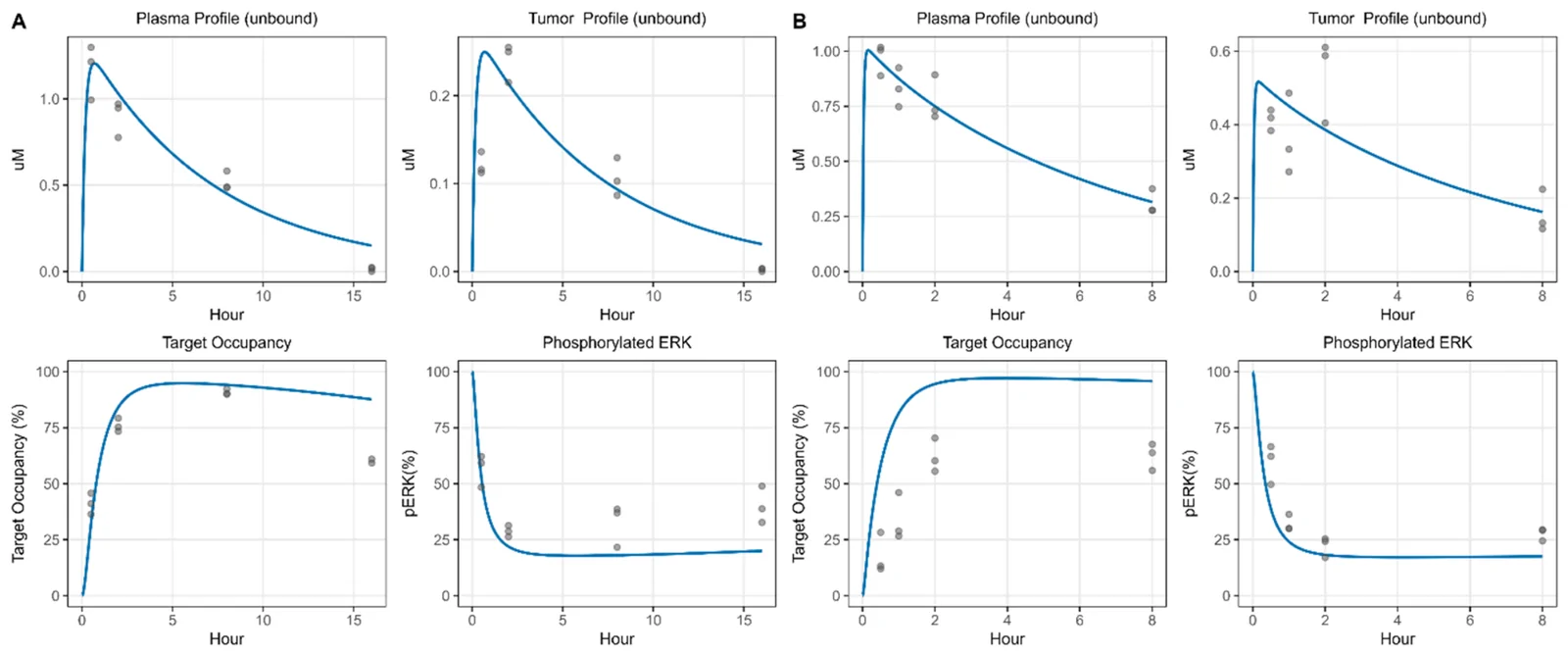
Pharmacodynamic model validation for two internal Amgen KRAS G12C inhibitors, Inhibitor-1 (**A**) and Inhibitor-2 (**B**). Plasma concentrations were fitted either with a one- or two-compartmental model, while tumor concentrations were predicted based on experimental tumor-plasma K_p,uu_. The PD model was then used to independently predict target occupancy (%TO) and p-ERK inhibition (%p-ERK). Red lines are model predictions and black circles are observations.

**Figure 5:**
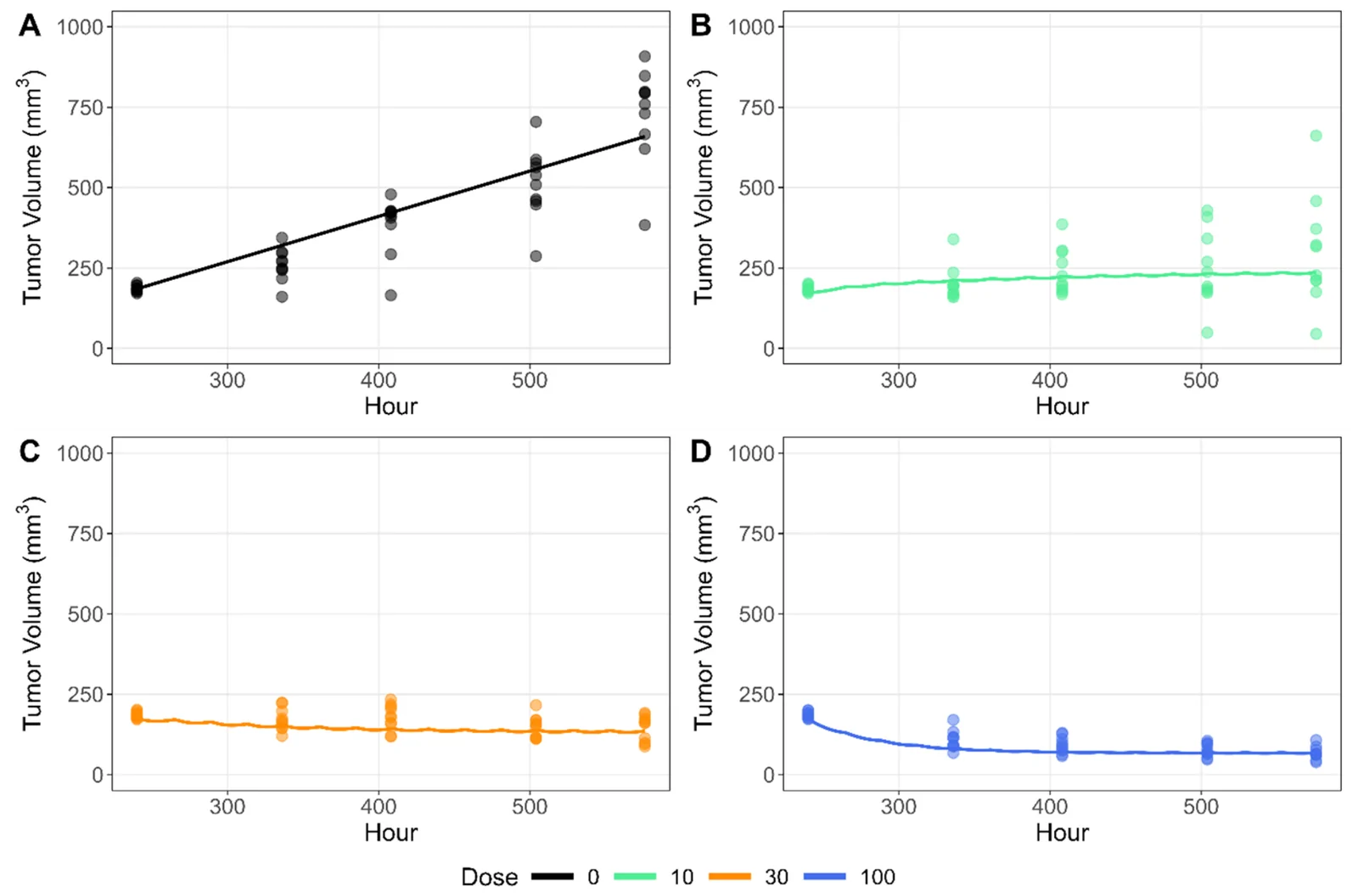
Validation of the efficacy model for an internal Amgen KRAS G12C inhibitor (inhibitor 1) following daily administration of 0 mg/kg (**A**), 10 mg/kg (**B**), 30 mg/kg (**C**), or 100 mg/kg (**D**) in mice bearing Mia PaCa-2 xenografts. Solid lines are model predictions, and solid circles are experimental observations. The zero-order tumor growth rate was estimated separately for each study on account of varying tumor growth kinetics across experiments.

**Figure 6:**
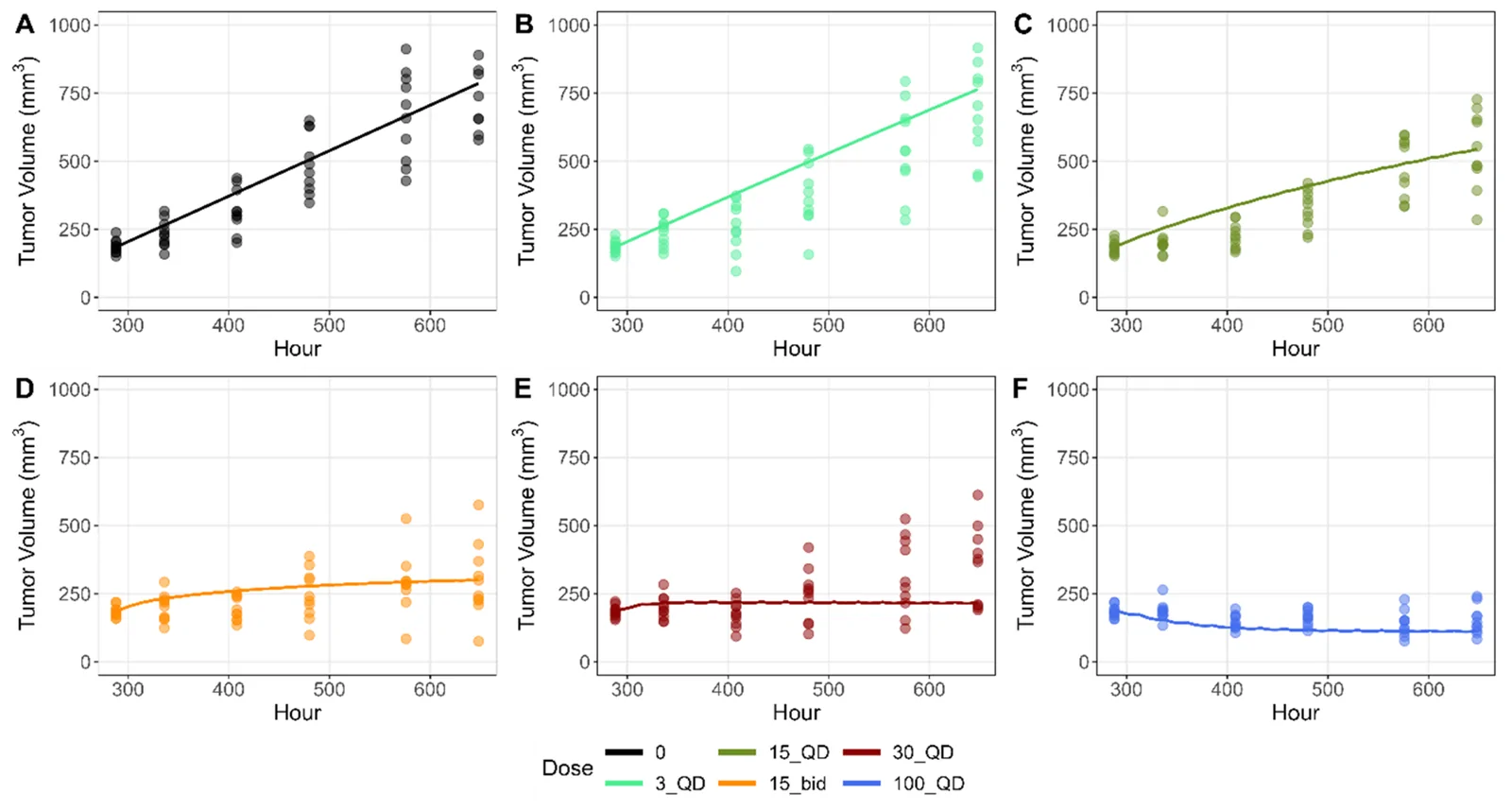
Validation of the efficacy model for an internal Amgen KRAS G12C inhibitor (inhibitor 2) following daily administration of 0 mg/kg (**A**), QD 3 mg/kg (**B**), QD 15 mg/kg (**C**), BID 15 mg/kg (**D**), QD 30 mg/kg (**E**), or QD 100 mg/kg (**F**) in mice bearing Mia PaCa-2 xenografts. Solid lines are model predictions, and solid circles are experimental observations. The zero-order tumor growth rate was estimated separately for each study given varying tumor growth kinetics across experiments.

Figure 4A,B show both PK and PD along with model-predicted simulations for two additional Amgen compounds. Once the exposure of these compounds was captured, the model was able to predict the time course of both TO and p-ERK in mouse xenografts. Figure 5 and Figure 6 show experimental observations and model predictions for tumor growth inhibition for the same two compounds for multiple dose levels. The model was successful in predicting tumor growth inhibition or regression in these studies, informing on the robustness of our model to predict PD and efficacy for multiple KRAS inhibitors.

Additionally, the model was also tested for three diverse classes of covalent inhibitors for KRAS G12C protein (non-Amgen compounds). The data for these compounds were obtained from publicly available sources; therefore, only limited information was available for model verification. For compound X1, the plasma concentrations were predicted based on the published PK parameters, and tumor-to-plasma K_P,uu_ was adjusted to capture observed TO. Once the TO was predicted correctly, the model was able to predict p-ERK and tumor growth inhibition reasonably well (Figure S1A). Similar to compound X1, PK for compound X2 was predicted based on published PK parameters. Tumor-to-plasma K_P,uu_ was assumed to be 1 for X2, which allowed the prediction of TO in the observation. Subsequently, the model was able to predict tumor growth inhibition in mice xenograft following 30 mg kg^−1^ QD dosing (Figure S1B). Similarly, for compound X3, tumor-to-plasma K_Puu_ was adjusted so that the prediction for p-ERK matched with observation. Subsequently, the model was able to predict tumor growth inhibition at 10 and 30 mg kg^−1^ (Figure S1C).

### 2.4. Results for OSM PK/PD Model

**Figure 7:**
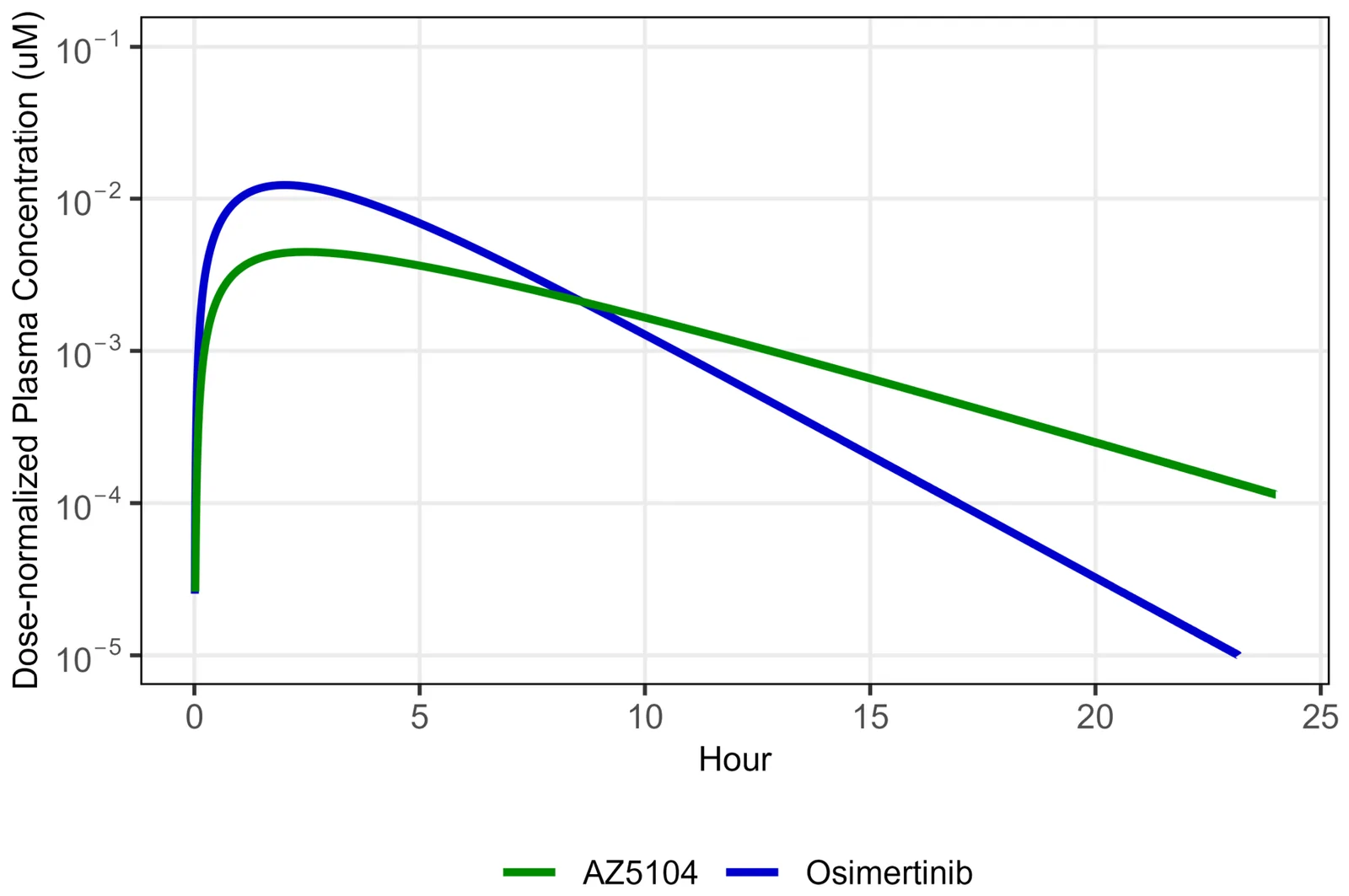
OSM PK model plasma concentration predictions in nude mice.

The OSM PK model predicted the dose-normalized PK profiles of OSM and AZ5104 reasonably well. There was a slight over-prediction of the PK profiles for both OSM and AZ5104 (Figure 7) with the reported equations and parameters.

The results for OSM p-EGFR and tumor volume reduction are provided in Table 2.

**Figure 8:**
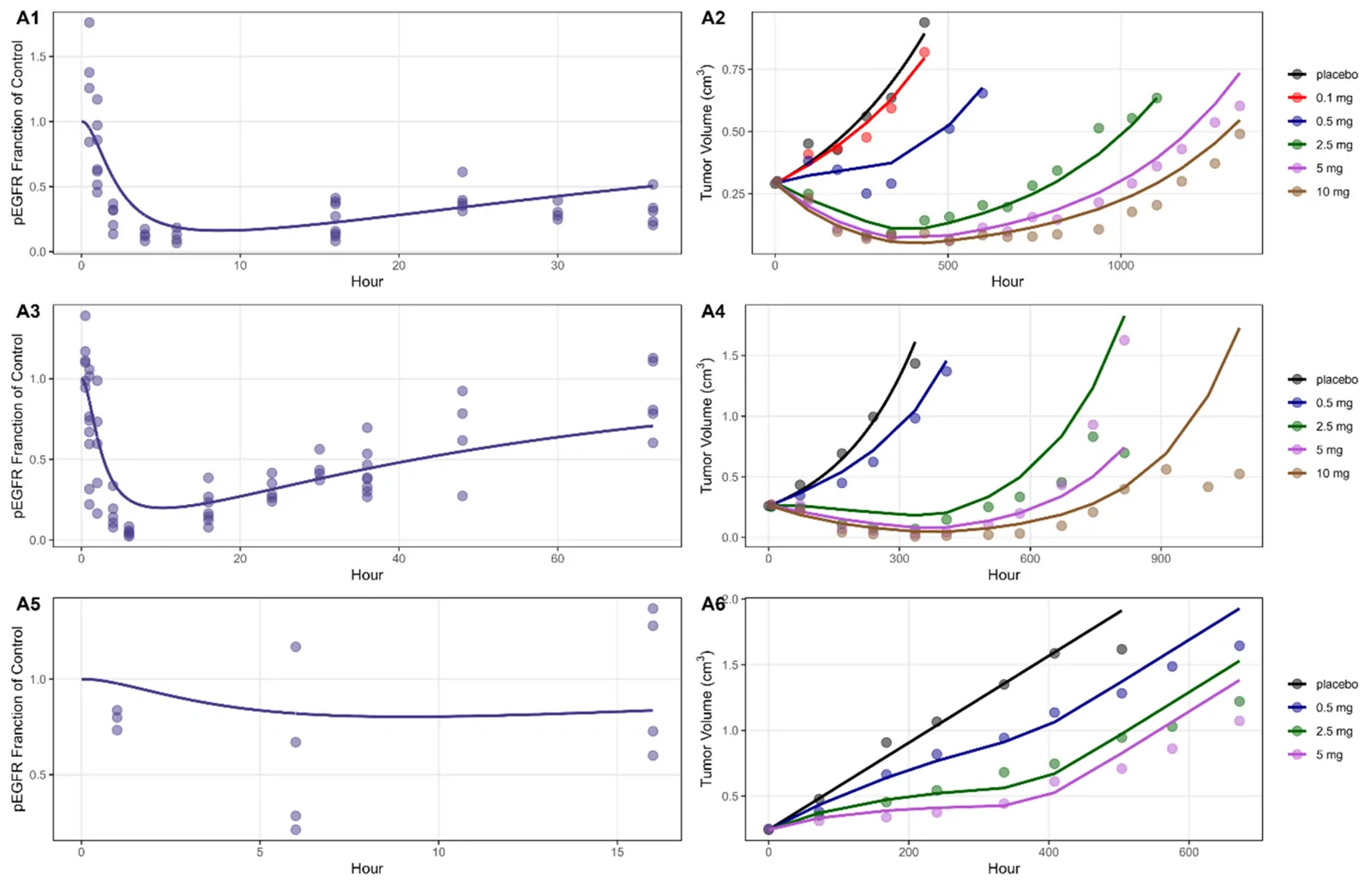
Model prediction and measured data for pEGFR (after 5 mg/kg dose) and tumor volume in PC9 (**A1**,**A2**), NCI-H1975 (**A3**,**A4**) and A431 (**A5**,**A6**).

Figure 8(A1) shows the PD fitting for the PC9 cell line. K_on_ was estimated to be 104 µM^−1^ h^−1^. There was a slight under prediction of p-EGFR at the earlier time points, but overall, the predictions were reasonable as compared to the measured p-EGFR. Figure 8(A2) shows the tumor growth curve fitting for the PC9 cell line for the placebo- and OSM-dosed groups. The first-order growth rate constant, k_g_, was estimated to be 2.606 × 10^−3^. The first-order tumor kill rate constant and hill co-efficient, nhill, were estimated to be 9.035 × 10^−3^ and 1.373, respectively, and the predicted tumor volume reduction reasonably matched the observed data.

For the NCI-H1975 cell line, k_on_ was estimated to be 79.4 µM^−1^ h^−1^ (Figure 8(A3)). Similar to PC9 cell line, there was a slight under-prediction of p-EGFR at the earlier time points, but overall, the predictions were reasonable as compared to the measured p-EGFR. Figure 8(A4) depicts the tumor growth curve fitting for the H1975 cell line for placebo- and OSM-dosed groups. The first-order growth rate constant, k_g_, was estimated to be 5.443 × 10^−03^. The first-order tumor kill rate constant and hill co-efficient, nhill, were estimated to be 0.0134 and 2.1082, respectively, and the predicted tumor volume reduction reasonably predicted the observed data.

Figure 8(A5) shows the p-EGFR fitting for the A431 cell line. K_on_ was estimated to be 1000 µM^−1^ h^−1^. The model under-predicted the observed data; OSM and AZ5104 did not effectively inhibit p-EGFR as seen in the observed data. Figure 8(A6) shows the tumor growth fitting for the A431 cell line for placebo and OSM dose groups. The zero-order growth rate constant, k_g_, was estimated to be 3.309 × 10^−03^. The first-order tumor kill rate constant and hill co-efficient, nhill, were estimated to be 0.0194 and 0.5961, respectively, and the predicted tumor volume reduction reasonably matched the observed data.

## 3. Discussion

The objective of this work was to establish a general modeling framework that could predict TO and change pharmacological biomarkers such as p-ERK and xenograft tumor growth inhibition following the administration of covalent inhibitors. The model utilized both compound/drug-related parameters (i.e., PK parameters, K_P,uu_, K_I_, k_inact_) and system-specific parameters (i.e., protein half-lives, concentrations etc.). This semi-mechanistic model only requires drug-related parameters for the prediction of PD and tumor growth inhibition if the system parameters are known. The half-life of KRAS G12C protein was reported to be 20–24 h. The absolute concentration of KRAS G12C protein in MIA PaCa-2 xenograft tumor was not known at the time when this modeling framework was developed. We initially estimated KRAS G12C protein concentration in MIA PaCa-2 xenograft as 0.0023 µM, and protein concentrations were subsequently determined by LC-MS/MS to be 0.0017 µM (data on file). Therefore, in the model, the KRAS G12C protein concentration was kept as 0.002 µM. The other two system parameters are related to p-ERK. Since the observed data for p-ERK inhibition was in percentage rather than absolute values, the baseline concentration of p-ERK was 100 in the model. The dephosphorylation of ERK1/2 is very fast and can happen within minutes [8]. Fujioka et al. reported that p-ERK half-life in RAS/ERK MAPK signaling cascade was 55 s, determined in a living cell experiment utilizing fluorescent probes [9]. Based on in vitro inhibition studies, we estimated the p-ERK half-life in HCC827 cells to be around 5 min (Amgen data on file). Subsequently, a p-ERK half-life of 0.1 h (~5 min) was used in the final model.

As shown in the results section, the model developed from publicly available sotorasib data was able to capture PD and TGI following the administration of KRAS G12C covalent inhibitors in xenograft mice. The model successfully predicted PD and efficacy for internal Amgen compounds as well as three non-Amgen compounds with diverse chemotypes, highlighting its applicability across distinct classes of covalent inhibitors. The model was subsequently applied for compound prioritization and human dose projection (HDP) efforts. However, the model under-predicted the TO of several highly potent inhibitors. For those compounds, k_on_ (the rate of initial non-covalent binding of the inhibitor to KRAS G12C protein) was faster than that of sotorasib and thus required compound-specific estimation. Once k_on_ was estimated, the model accurately predicted both p-ERK and TGI for highly potent inhibitors (Supplementary Figures S2 and S3).

In addition to KRAS G12C covalent inhibitors, the modeling framework was extended to EGFR covalent inhibitors. AZ5104 and OSM contributed to the inhibition of p-EGFR. Initially, the objective was to build a simple PK model for parent and metabolite to describe OSM and AZ5104. A simple PK model could not be fitted to the data published in Figure 2c,D in Yates et al. Thus the PK model published in Yates et al. was reproduced with the published equations and model parameters (Equations (S1)–(S32) in the Supplementary Materials). The irreversible model was then merged with the PK model (Equations (S7)–(S14)) to predict the inhibition of p-EGFR and TGI. The simulated OSM and AZ5104 concentration was slightly over-predicted after using the published equations and parameters, but the shapes of the PK profiles of OSM and AZ5104 were the same as those published by Yates et al. (Figure 6). An assumed high value of K_T_ was to ensure that plasma T_max_ was equal to tumor T_max_. The k_inact_ value for OSM was obtained from Zhai et al. [10]. Since the potency of an irreversible inhibitor is described by k_inact_, the relative potency for AZ5104 was multiplied by the k_inact_ of AZ5104 to describe the greater potency of AZ5104 as compared to OSM. EGFR, being a membrane receptor, does not have a measured concentration value reported in the literature. Yates et al. used a baseline value of p-EGFR of 100%. For our binding model, a concentration value is required. Thus, we assumed p-EGFR value to be similar to KRAS_F_ (0.002 µM).

The p-EGFR fittings for all cell lines were reasonable; however there were some under-predictions at the earlier time points. This could be due to the in vitro measured k_inact_ value obtained from the literature. The model published by Yates et al. performed better in fitting the earlier p-EGFR measurements. This could be because Yates et al. fitted all the parameters of their PD model. We fitted K_on_ in the PD model and fixed kinact to the value obtained from Zhai et al. [10], but in vivo, OSM and AZ5104 could be more potent. k_inact_ was not fitted, since our objective was to show that tumor growth inhibition could be predicted by in vitro measured k_inact_ values. Another reason for under-prediction could be due to the fitting of digitized data and the accuracy with which this data was digitized. The identifiability issue of parameters from the A431 cell line is potentially due to a lack of pEGFR inhibition and the high variability in the measurements. This was not the issue for H175 and PC9 cell lines, where more dense measurements were available. The tumor growth rate constants were fitted for each cell line since different cell lines have different growth kinetics. The compound-treated dose groups were also fitted to obtain k_kill_ and nhill. k_kill_ and nhill fitted from one cell line did not predict tumor growth inhibition for other cell lines. Yates et al. fitted N (power term on Fkill) for each cell line, similarly to k_kill_ and nhill in our model. Due to the differences in the structures of the two models, we were required to fit both k_kill_ and nhill. The authors acknowledge that the EGRF modeling framework was adaptable to EGFR covalent inhibitors but required target- and cell-line-specific parameterization. However, k_kill_ and nhill were estimated due to different kill kinetics of the three cell lines. With the KRAS model, we showed that k_kill_ and nhill can be fitted from one study and then used to simulate tumor growth inhibition for other studies when the cell line was the same.

The OSM and AZ5104 PK-PD model structure could be modified to separate EGFR_I_ into separate compartments forming from OSM and AZ5104. The model was modified into two compartments, EGFR_I_P_ and EGFR_I_M_, and EGFR and tumor growth inhibition data were refitted. The visual fits, parameter estimates and CV(%) were the same as without the modified model i.e., within 5%. Thus, the lumped EGFR_I_ compartment was used for this publication. Further work is required to understand for which parent and metabolite combined effect; it is required to have EGFR_I_ formed separately from the parent and metabolite.

Several PK/PD models for covalent inhibitors have been published previously. Multiple mechanistic PK/PD models have been published for BTK inhibitors. A mechanistic PK/PD model for CC-292 was developed by Daryaee et al., using both in vitro and in vivo data and applying the framework established for *P. aeruginosa* LpxC inhibitors [11]. The model included a two-step binding mechanism and accounted for competition between the drug and ATP for the target. The model revealed that a high level of TO is needed to achieve a desirable outcome of CC-292 [12]. Meng et al. developed a semi-mechanistic PK/PD model using phase 1 data for tirabrutinib. The model fitted free and total BTK using separate equations rather than adopting mechanistic binding equations. A feedback component was incorporated into the model to account for the elevated total BTK levels observed following the repeated administration of tirabrutinib. The model was able to adequately capture both free and total BTK levels observed in the phase 1 study for tirabrutinib [13]. More recently, Demin Jr. et al. developed a mechanistic model for covalent BTK inhibitors. The model employed two-step binding equations similar to those used in this approach while also incorporating the competitive binding of ATP to BTK. The model was validated against clinical BTK TO for acalabrutinib, ibrutinib, and zanubrutinib in peripheral blood mononuclear cells (PBMC) and lymph nodes. Following validations, the model was applied to predict BTK TO in PBMCs, lymph nodes, and bone marrow under various clinical scenarios, including temporary dose reductions due to toxicity and missed doses [14].

This modeling framework employs the same two-step binding scheme as the above models to describe target occupancy (TO). However, beyond TO prediction, our framework is capable of predicting a downstream biomarker (p-ERK) and tumor growth inhibition based on covalent inhibitor–target engagement. This framework was also able to predict TO, p-ERK, and xenograft tumor growth inhibition of KRAS G12C covalent inhibitors based solely on compound PK and in vitro parameters. Additionally, we demonstrate that this framework can be broadly applied to predict pharmacodynamic responses and efficacy of EGFR covalent inhibitors.

**Figure 9:**
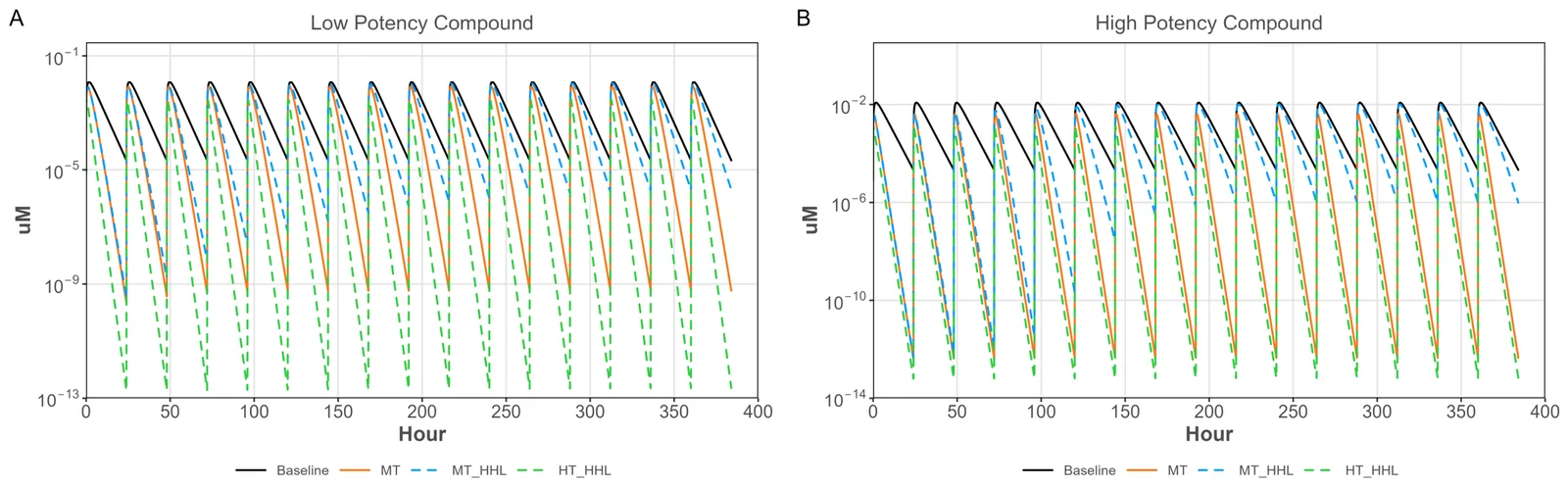
Effects of target protein pool and half-life on the exposure of covalent inhibitors. (**A**) Low-potency inhibitors (K_I_ = 100 µM, k_inact_ = 3000 h^−1^, k_on_ = 10 µM^−1^h^−1^) and (**B**) high-potency inhibitors (K_I_ = 100 µM, k_inact_ = 30,000 h^−1^, k_on_ = 50 µM^−1^h^−1^). Legends—MT: Target pool increased by 20,000-fold, MT_HHL: Target pool increased by 20,000-fold and half-life increased by 10-fold, HT_HHL: Target pool increased by 200,000-fold and half-life increased by 10-fold.

Mechanistic PK/PD modeling is applied in early development to inform compound selection and to predict biomarkers of interest and HDP. Consequently, these models are useful for identifying the extent to which model parameters influence pharmacodynamic outcomes or HDP. Yang described a PK/PD model for covalent inhibitors and investigated the effect of drug clearance, inactivation potency, target expression level, and target turnover half-life on HDP [5]. Their work demonstrated that target turnover half-life is a key determinant of human dose. Short target half-lives necessitate more frequent dosing due to rapid target resynthesis, whereas long target half-lives limit target recovery prior to subsequent dosing. On the other hand, the impact of target expression levels on human dose is more prominent for low clearance drugs than for high clearance drugs due to the potential for target-mediated drug disposition (TMDD). Daryaee et al. carried out similar simulation efforts and concluded that, to maintain appreciable TO for targets with shorter turnover half-life, drugs need to have high affinity, a slow dissociation rate, and low clearance [4]. Figure 9 shows the effects of target protein pool and half-life on the clearance of high- and low-potency covalent inhibitors. A substantial target protein pool was required to produce appreciable TMDD in the xenograft model. The 20,000- and 200,000-fold increases in target abundance represent hypothetical scenarios selected to illustrate the conditions under which TMDD begins to meaningfully affect plasma exposure. Figure 9A shows the impact of exposure of a low-potency compound (K_I_ = 100 µM, k_inact_ = 3000 h^−1^, k_on_ = 10 µM^−1^h^−1^) if the target pool in xenograft was increased by 20,000-fold. The reduction in exposure due to TMDD could be recovered with multiple dosing when the target protein half-life was substantially increased (10-fold in the simulation), as a longer half-life allows target protein saturation to be achieved following repeated dosing. When the target protein pool was increased 200,000-fold, even a 10-fold increase in target half-life was insufficient to restore exposure to baseline levels. This reflects insufficient inhibitor availability to saturate the very large target pool; under these conditions, an increase in inhibitor dose would be required to achieve target saturation. The reduction in exposure due to TMDD for a high-potency compound (K_I_ = 100 µM, k_inact_ = 30,000 h^−1^, k_on_ = 50 µM^−1^h^−1^), was more pronounced. As shown in Figure 9B, both 20,000- and 200,000-fold increases in target protein pool resulted in similar reductions in exposure. However, exposure could be recovered with multiple dosing when the target protein half-life was increased 10-fold for the 20,000-fold increase in target pool, but not for the 200,000-fold increase. Overall, TMDD in a covalent inhibitor is unlikely to be observed when the target is confined to the xenograft tumor. In contrast, TMDD may become apparent when the target is expressed extensively outside the tumor compartment, such as in systemic circulation.

**Figure 10:**
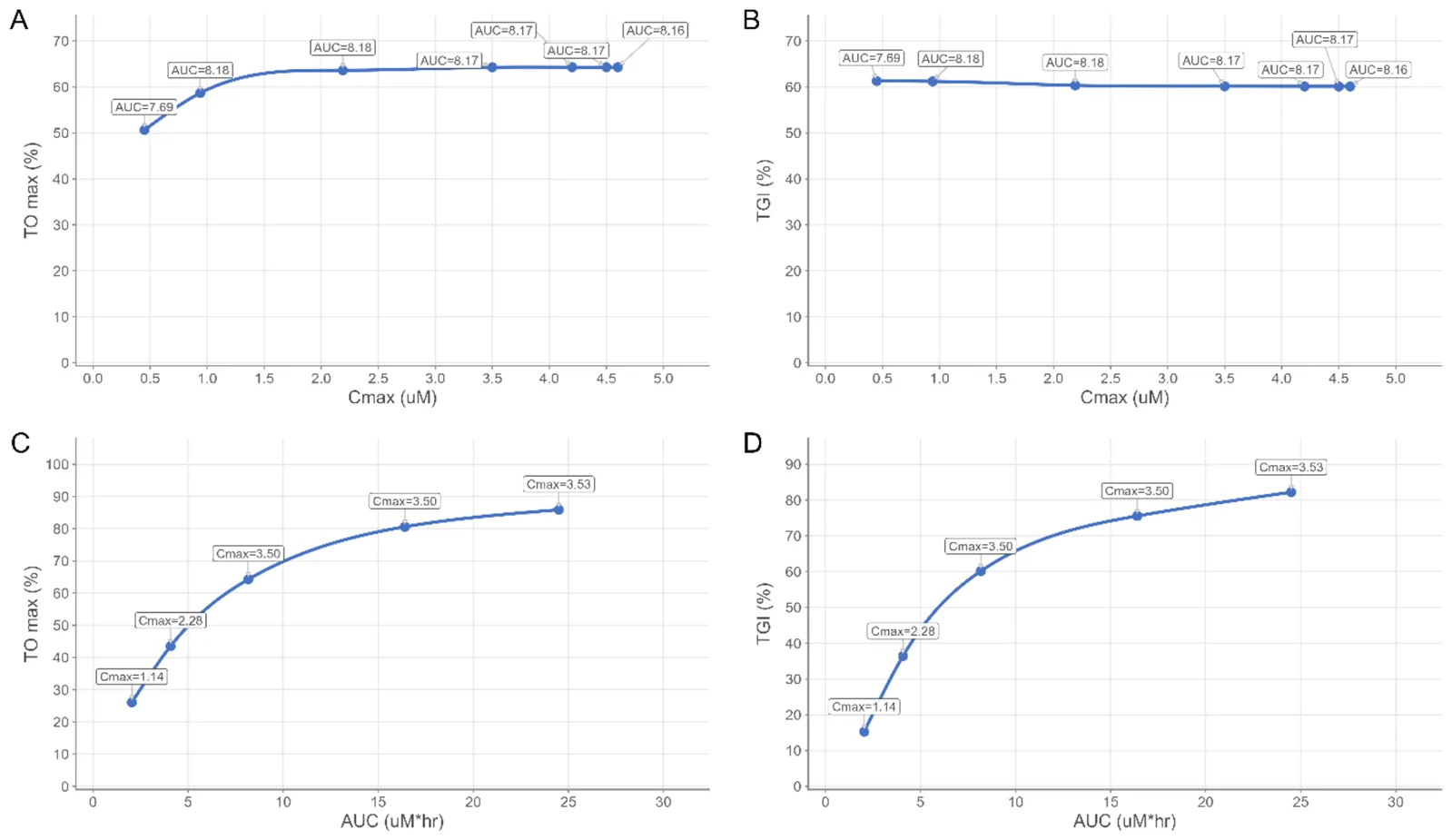
Sensitivity analysis showing the effects of C_max_ on target occupancy (%TO; (**A**)) and tumor growth inhibition (TGI; (**B**)), and the effects of AUC on %TO (**C**) and TGI (**D**) for KRAS G12C inhibitors.

One advantage of covalent drugs is that sustained exposure above a specific threshold (e.g., IC_90,u_) is not required to achieve the desired level of target inhibition. Once the target is covalently inhibited, the associated signaling pathway remains suppressed until new target proteins are resynthesized. Consequently, for covalent drugs, C_max_ may appear to be a more critical pharmacokinetic parameter than AUC upon initial assessment. Based on simulations using their model for covalent inhibition, Strelow identified AUC as the primary pharmacokinetic parameter driving target occupancy [6]. Consistent with this, Weiss et al. later showed that anti-tumor efficacy depends on the AUC of JDQ443, a covalent KRAS G12C inhibitor [15]. In order to investigate further, we performed simulations to evaluate the impact of both the C_max_ and AUC of KRAS G12C inhibitors on xenograft tumor TO and TGI. First, simulations were carried out by fixing all of the model parameters and only varying k_a_ to achieve different values of C_max_. As shown in Figure 10A,B, increasing C_max_ from 0.45 to 3.5 µM resulted in a modest increase in peak TO from 50% to 64%, with no further increase in TO observed at higher C_max_ values. In a second set of simulations, both dose and k_a_ were varied to achieve increasing AUC while maintaining C_max_ at approximately the same level. As shown in Figure 10C,D, there was a significant effect of AUC on both TO and TGI (increasing AUC from 2 to 24.5 µM · h resulted in an increase in peak TO from 26% to 85% and TGI from 15% to 82%). AUC showed a stronger association with sustained target occupancy and tumor growth inhibition than C_max_.

Overall, the framework successfully linked drug exposure to TO, pERK, and tumor efficacy across multiple KRAS G12C inhibitors and was adaptable to EGFR covalent inhibitors, supporting its potential utility in covalent drug development. Nevertheless, several limitations should be considered when interpreting these findings: (a) non-specific binding of the covalent warhead was not accounted for in the model; (b) although the sotorasib plasma concentration–time profile was biphasic, a one-compartment PK model with the absorption rate constant fixed at 1 h^−1^ was used because the parameters of a two-compartment model were not identifiable, which may have introduced some bias into the estimated pharmacodynamic parameters; (c) the individual fitting of k_on_ was needed for highly potent covalent inhibitors; (d) because tumor concentration data were unavailable, the tumor-to-plasma kpuu was assumed to be 1 in some cases. Although the model adequately described the observed pharmacodynamic responses under this assumption, experimental tumor concentration data are needed to verify its validity. (e) Several parameters in the EGFR model were estimated with limited precision due to limited data; (f) data used for external validation were digitized from published figures and may therefore contain minor inaccuracies.

## 4. Method

### 4.1. Ethics Statement

Mice were housed in groups at an AAALAC, International-accredited facility. Animals were cared for in accordance with the Guide for the Care and Use of Laboratory Animals, 8th Edition. All research protocols were reviewed and approved by the Amgen Institutional Animal Care and Use Committee. All studies utilized 4–7-week-old female athymic nude mice (Charles River Laboratories). The athymic nude mice were housed in individual ventilated caging (IVC) system (Tecniplast greenline) on an irradiated corncob bedding (Envigo Teklad 7097, Inotivi Inc., West Lafayette, IN, USA). Lighting in animal holding rooms was maintained on a 12:12 h-light:dark cycle, and the ambient temperature and humidity range were at 68 to 79 F and 30 to 70%, respectively. Animals had ad libitum access to irradiated pelleted feed (Envigo Teklad Global Rodent Diet-soy protein free extruded, irradiated 2920X) and reverse-osmosis (RO)-chlorinated (2 to 3 ppm) water via an automatic watering system.

### 4.2. Cell Lines

MIA PaCa-2 cells used in the in vivo xenograft studies were purchased from the American Type Culture Collection (ATCC CRL-1420) and were passaged in mice to generate MIA PaCa-2 T2. Cells were authenticated by short tandem repeat (STR) profiling and tested negative for mycoplasma.

### 4.3. Data Source—Sotorasib

#### 4.3.1. In Vitro Data

The turnover half-life of KRAS G12C protein and potency parameters—k_inact_ (maximum rate of inhibition) and K_I_ (concentrations that achieve a half-maximal rate)—for sotorasib were taken from a publication by Canon et al. [7]. The expression level of KRAS G12C protein in MIA PaCa-2 cells was determined by liquid chromatography-tandem mass spectrometry (LC/MS/MS) assay.

For other Amgen internal KRAS G12C inhibitors, k_obs_/[I] was determined from biochemical assays (data on file). It was assumed that k_inact_/K_I_ is equivalent to k_obs_/[I], since the K_I_ for these compounds were around 100 µM and the unbound plasma concentrations were much lower than K_I_.

#### 4.3.2. In Vivo Data

All in vivo data for sotorasib were taken from a publication by Canon et al. [7]. For other Amgen internal KRAS G12C inhibitors, similar approaches were taken to determine plasma and tumor exposures, target occupancy (%TO), the phosphorylation of ERK (% p-ERK), and efficacy in MIA PaCa-2 tumor-bearing mice. In addition, comparable data from internal Amgen compounds were used for model validation. All associated in vivo experiments were conducted at Amgen facilities under protocols approved by the Amgen Institutional Animal Care and Use Committee (IACUC).

### 4.4. Model Development

The model was developed by sequentially fitting published PK, PD, and efficacy following oral administrations of 1, 3, 10, 30, and 100 mg kg^−1^ of sotorasib in MIA PaCa-2 xenograft-bearing mice. A one-compartment model was used to fit the unbound plasma PK of sotorasib. Subsequently, a tumor compartment was added to the model with a starting xenograft volume of 200 mm^3^. The transfer of the drug from plasma to tumor was considered to be rapid, and this was represented by setting the rate to an arbitrarily high value of 1000 L h^−1^. The unbound tumor concentrations of sotorasib were fitted by estimating unbound tumor to unbound plasma ratio (K_p,uu_).

A mechanistic two-step reaction for covalent inhibition (Equations (1) and (2)) was used to describe the target occupancy of sotorasib.

(1) $$\frac{{dKRAS}_{F}}{dt}=K_{syn}-(0.693/K{RAS}_{thalf})\times {KRAS}_{F}-K_{on}\times {KRAS}_{F}\times TUM/V_{T}+K_{off}\times {KRAS}_{I}$$

(2) $$\frac{{dKRAS}_{I}}{dt}=K_{on}\times {KRAS}_{F}\times \frac{TUM}{V_{T}}-K_{off}\times {KRAS}_{I}-K_{inact}\times {KRAS}_{I}-\left(0.693/K{RAS}_{thalf}\right)\times {KRAS}_{I}$$

(3) $$\frac{{dKRAS}_{B}}{dt}=K_{inact}\times {KRAS}_{I}-\left(0.693/K{RAS}_{thalf}\right)\times {KRAS}_{B}$$

where *KRAS_F_*, *KRAS_I_*, and *KRAS_B_*, represent the free, reversibly drug-bound, and covalently drug-bound KRAS G12C protein, respectively. *TUM* is the amount of drug in the tumor, and *V_T_* is the volume of the xenograft tumor. The turnover of *KRAS* G12C was modeled using the zero-order synthesis rate (k_syn_ = 0.693/KRAS_thalf_)× KRAS_0_ ×V_T_) and first-order degradation rate, where KRAS_thalf_ and KRAS_0_ are the degradation half-life and the concentration of protein in MIA PaCa-2 cells, respectively. k_inact_ is the first-order rate of inactivation of drug–protein complex and *K_I_ = k_off_/k_on_*; *k_on_* and *k_off_* represent the second-order association rate and first-order dissociation rate of drug–protein binding. Percent TO was calculated as follows:

(4) $$\%TO=(1-({KRAS}_{F}/({KRAS}_{F}+{KRAS}_{I}+{KRAS}_{B})))\times 100$$

The PD model for p-ERK is described by an indirect response model in which %TO inhibits the production of p-ERK, as illustrated in Equation (5).

(5) $$\frac{dp\text{-}ERK}{dt}=K_{in}\times (1-0.83997\times ({\left(TO/100\right)}^{nhill})-(0.693/{p\text{-}ERK}_{thalf})\times p\text{-}ERK)$$

*k_in_* is the zero-order production rate of *p-ERK* and is given by *k_in_* = (0.693/*p-ERK_thalf_*)× *p-ERK*_0_; *nhill* is a hill function; *p-ERK_thalf_* is the half-life of phosphorylated ERK. This equation allows a maximum *p-ERK* inhibition of ~83% based on the maximum inhibition observed for sotorasib.

Subsequently, the PD model was linked to a simple linear tumor growth model and a first-order tumor inhibition model driven by *p-ERK* inhibition:

(6) $$\frac{dT_{v}}{dt}=k_{g}-k_{kill}\times V_{T}\times {\left(1-\frac{p\text{-}ERK}{100}\right)}^{gamma}$$

where *V_T_* is the tumor volume, *k_g_* is the zero-order tumor growth rate, *k_kill_* is the first-order tumor inhibition rate, and *gamma* is the shape factor.

### 4.5. Model Validations

The mechanistic PK/PD–Efficacy model was validated using data from Amgen’s internal KRAS G12C inhibitors. The plasma and tumor profiles for each of these compounds were fitted first to derive the PK parameters. Once the PK parameters were estimated, simulations were carried out for *%TO*, *% p-ERK*, and tumor growth inhibition/regression based solely on the exposure and potency (*k_inact_* and *K_I_*) of each of the compounds. Furthermore, the model’s performance was also assessed by comparing its predictions for *%TO* and efficacy against published data for several KRAS G12C covalent inhibitors.

### 4.6. Application of the Model to EGFR Inhibitor Osimertinib (OSM)

#### 4.6.1. In Vivo Data

In vivo data for OSM were obtained from publication by Yates et al. [16]. They had published the pharmacokinetics of OSM and its metabolite, AZ5104, p-EGFR reduction after the administration of osimertinib in various cell lines, and the growth reduction of tumor xenografts of the same cell lines in female severe combined immune-compromised (CB17 scid) mice (PC9 xenografts) and female athymic (nu/nu:Alpk) mice (NCIH1975 and A431 xenografts).

#### 4.6.2. Method for OSM

The OSM model was adapted from the equations and model parameters published in the supplementary material of Yates et. al. [16]. The OSM PD and efficacy model equations are given below:

(7) $$\frac{d{TUM}_{P}}{dt}=K_{T}\times \left(\frac{{CEN}_{P}\times f_{u,p}}{V_{1,p}}-\left(\frac{\frac{{TUM}_{P}}{V_{T}}}{K_{P}}\right)\right)-K_{on,P}\times {EGFR}_{F}\times \frac{{TUM}_{P}}{V_{T}}+K_{off,P}\times {EGFR}_{I}$$

(8) $$\frac{d{TUM}_{M}}{dt}=K_{T}\times \left(\frac{{CEN}_{M}\times f_{u,M}}{V_{1,M}}-\left(\frac{\frac{{TUM}_{M}}{V_{T}}}{K_{P}}\right)\right)-K_{on,M}\times {EGFR}_{F}\times \frac{{TUM}_{M}}{V_{T}}+K_{off,M}\times {EGFR}_{I}$$

(9) $$\frac{d{EGFR}_{F}}{dt}=K_{syn}-(\frac{0.693}{{EGFR}_{thalf}})\times {EGFR}_{F}-K_{on,P}\times {EGFR}_{F}\times \frac{{TUM}_{P}}{V_{T}}+K_{Off,P}\times {EGFR}_{I}\;-K_{on,M}\times {EGFR}_{F}\times \frac{{TUM}_{M}}{V_{T}}+K_{off,M}\times {EGFR}_{I}$$

(10) $$\frac{d{EGFR}_{I}}{dt}=K_{on,P}\times {EGFR}_{F}\times \frac{{TUM}_{P}}{V_{T}}-K_{off,P}\times {EGFR}_{I}+K_{on,M}\times {EGFR}_{F}\times \frac{{TUM}_{M}}{V_{T}}-K_{off,M}\times {EGFR}_{I}\;-K_{inact,P}\times {EGFR}_{I}-K_{inact,M}\times {EGFR}_{I}-(\frac{0.693}{{EGFR}_{thalf}})\times {EGFR}_{I}$$

(11) $$\frac{d{EGFR}_{B}}{dt}=K_{inact,P}\times {EGFR}_{I}+K_{inact,M}\times {EGFR}_{I}-(\frac{0.693}{{EGFR}_{thalf}})\times {EGFR}_{B}$$

(12) $$\%TO=\left(1-\frac{{EGFR}_{F}}{{EGFR}_{F}+{EGFR}_{I}+{EGFR}_{B}}\right)\times 100$$

(13) $$\frac{d{TUM}_{V}}{dt}=k_{g}\times {TUM}_{V}-k_{kill}\times {TUM}_{V}\times {\left(\frac{TO}{100}\right)}^{nhill}$$

where CEN_P_ and CEN_M_ are the plasma concentration of OSM and AZ5104, respectively. Figure 2c from Yates et al. [16] was reproduced to validate the model. Figure 7 depicts the reproduced Figure 2c from Yates et al. The objective was to reproduce Figure 4 (A1, A2, B1, B2, C1, C2) of Yates et al. with the model described in Section 2.2 for KRAS inhibitors for irreversible inhibitors. Tumor-to-plasma *K_p,uu_* for OSM was assumed to be 1. Given the empirical tumor PK model, a tumor-to-plasma *K_p,uu_* of 1 indicates a free plasma concentration equal to free tumor concentration. The *p-EGFR* turnover rate for each cell line was obtained from the Supplementary Table 1 of Yates et al. [16] It is reported that the metabolite of OSM, AZ5104, was more potent than OSM, and Yates et al. [16] reported relative potency of AZ5104 for each cell line. In our model, the relative potency of AZ5104 was multiplied by *k_inact_* to account for the relative potencies of OSM and AZ5104. The concentration of free p-EGFR was assumed to be equal to free KRAS protein. *K_on_* was assumed to be the same for OSM and AZ5104, since the relative potency of the metabolite was managed by multiplying with *k_inact_*. *p-EGFR*, and tumor volume data was digitized [17] from Figure 4 of Yates et. al. Similar to the KRAS model described above for high-potency compounds, the objective was to fit the *p-EGFR* data with the PK-irreversible model and estimate *K_on_*. The fitted *K_on_* was used to predict the tumor volume data. The tumor volume data from the placebo groups were fitted with a simple exponential growth model for PC9 and H1975. For the A431 model, the placebo tumor volume was fitted with a zero-order growth curve. For the tumor volumes in the treated groups, the initial objective was to fit one cell line to estimate the *k_kill_* and nhill parameter and fix the *k_kill_* parameter, but estimate *nhill* for the other cell lines. This method resulted in the poor prediction of tumor growth regression. Hence, *k_kill_* and *nhill* was estimated for all cell lines.

## 5. Conclusions

Mechanistic PK/PD models utilize mathematical equations to describe the mechanistic interactions of drugs and their targets. Once a mechanistic model is established and validated, this model can independently predict the PD/TGI of compounds if the drug-related parameters are known. Due to such models’ mechanistic nature, they can be translated across species and extrapolated to different scenarios (e.g., co-administration of a second inhibitor). In this work, we developed a mechanistic PK/PD–efficacy modeling framework for KRAS G12C covalent inhibitors. This model was able to independently predict TO, p-ERK levels, and efficacy in tumor xenografts using only the PK curves and potency of covalent inhibitors. Moreover, we demonstrated that this same modeling framework could be extended to predict pharmacodynamic responses for EGFR inhibitors.

## Figures and Tables

**Table 1:** Model parameters for KRAS G12C inhibitors.

Parameter	Unit	Estimate (%CV)
ka	1/h	1 (fixed)
Vc_u_	L/kg	45.82 (5.67)
CL_u_	L/h/kg	5.41 (79.79)
vtumor	mm^3^	200
kpuu	-	1.08 (6.64)
Kinact	1/h	3060
KI	uM	86
KRAS_0_	uM	0.002
KRAS_thalf_	h	22
kon	1/h·μM	10.56 (12.77)
koff	1/h	908.16
pERK_0_	%	100
pERK_thalf_	h	0.1
nhill	-	0.3471 (14.24)
kg	mm^3^/h	1.75 (5.66)
kkill	1/h	0.088 (32.68)
Log gamma	-	0.81 (8.46)

**Table 2:** pEGFR and tumor volume model parameters for PC9, NCI-H1975 and A431 cell lines.

Cell Line	Parameter	Unit	Estimate (% CV)
PC9	k_on_	1/h·μM	104 (54)
	K_g_	1/h	2.606 × 10^−3^ (27)
	k_kill_	1/h	9.035 × 10^−3^ (27)
	nhill	-	1.373 (54)
NCI-H1975	k_on_	1/h·μM	79 (39)
	K_g_	1/h	5.443 × 10^−3^ (30)
	k_kill_	1/h	0.0134 (65)
	nhill	-	2.1082 (106)
A431	k_on_	1/h·μM	1000 (>1000)
	K_g_	mm^3^/h	3.309 × 10^−3^ (4)
	k_kill_	1/h	0.0194 (32)
	nhill	-	0.5961 (20)

## Data Availability

The datasets presented in this article are not publicly available, as they contain proprietary information owned by Amgen Inc. Requests for access to the datasets should be directed to udahal@amgen.com.

## Supplementary Materials

The following supporting information can be downloaded at: https://www.mdpi.com/article/10.3390/ph19081228/s1, Figure S1: Model validation using literature-reported pharmacodynamic and tumor growth efficacy data for three KRAS G12C inhibitors. Solid lines are model predictions and the solid circles are published experimental observations; Figure S2: Target occupancy (solid circles) for a high potency Amgen internal KRAS G12C covalent inhibitor (compound H1). The model was fitted (solid line) to estimate K_on_ which was estimated to be 51.31 1/hr·µM; Figure S3: Tumor growth inhibition efficacy simulation for compound H1. K_on_ = 51.31 1/hr·µM was used of all of the simulations; OSM and AZ5104 PK Model Equations (S1)–(S32); Sotorasib PK Model Equations (S33)–(S35).
