# Supplementary material for: Development of a PK/PD–Efficacy Modeling Framework for Covalent Inhibitors
Source: Pharmaceuticals (Basel). 2026 Aug 4;19(8):1228. doi: 10.3390/ph19081228 (PMC13516021; doi:10.3390/ph19081228)
Supplement: Supplementary file 1 [file pharmaceuticals-19-01228-s001.zip › pharmaceuticals-4405088-supplementary.pdf]

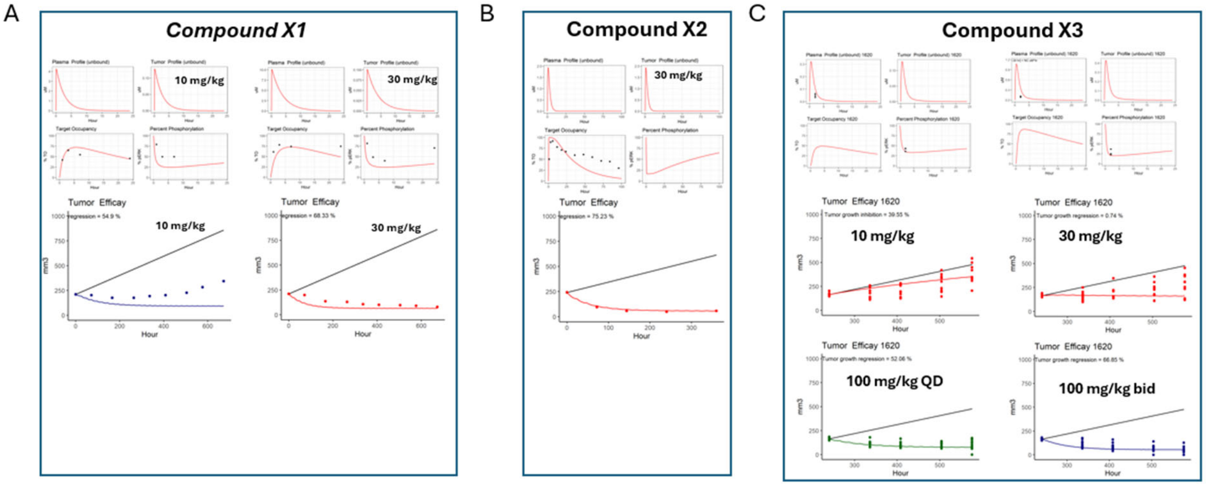

Figure S1: Model validation using literature-reported pharmacodynamic and tumor growth efficacy data for three KRAS G12C inhibitors. Solid lines are model predictions and the solid circles are published experimental observations.

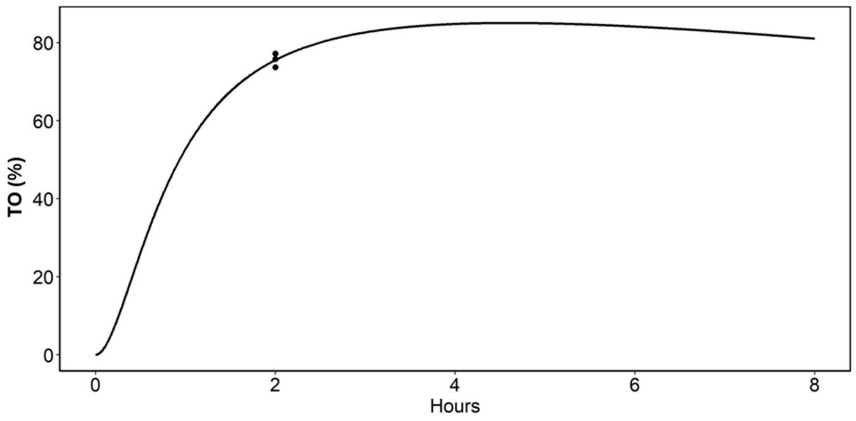

Figure s2 - Target occupancy (solid circles) for a high potency Amgen internal KRAS G12C covalent inhibitor (compound H1). The model was fitted (solid line) to estimate K_{on} which was estimated to be 51.31 1/hr · µM.

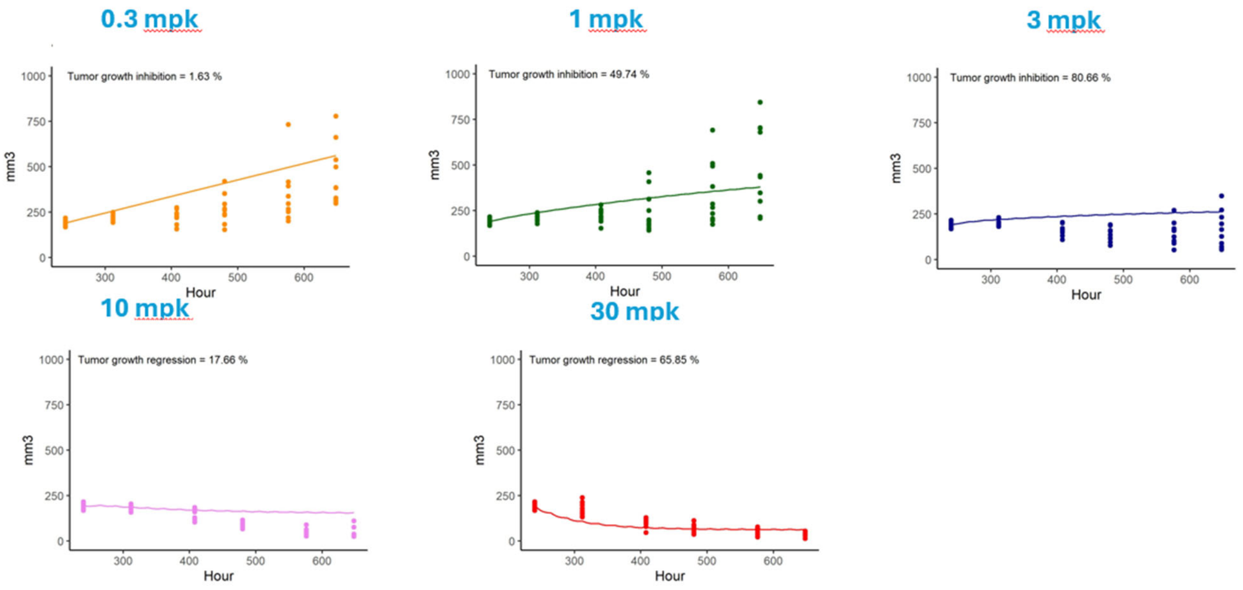

Figure s3 – Tumor growth inhibition efficacy simulation for compound H1. K_{on} = 51.31 1/hr · µM was used of all of the simulations.

OSM and AZ5104 PK Model Equations

$$f_{GUT1P,GUT2P} = Fab_P \times \left(1 - \frac{CL_P}{Q_H}\right) \cdots S1$$

$$f_{GUT1P,GUT2M} = Fab_P \times \left(\frac{CL_P}{Q_H}\right) \times f_{MET} \cdots S2$$

$$f_{GUT1P,LOST} = 1 - f_{GUT1P,GUT2P} - f_{GUT1P,GUT2M} \cdots S3$$

$$f_{GUT1M,GUT2M} = Fab_M \times \left(1 - \frac{CL_M}{Q_H}\right) \cdots S4$$

$$f_{GUT1M,LOST} = 1 - f_{GUT1M,GUT2M} \cdots S5$$

$$V_{2,P} = V_{SS,P} - V_{1,P} \cdots S6$$

$$V_{2,M} = V_{SS,M} - V_{1,M} \cdots S7$$

$$J_{GUT1P,LOST} = f_{GUT1P,LOST} \times k_{ab,P} \times GUT_{1,P} \cdots S8$$

$$J_{GUT1P,GUT2P} = f_{GUT1P,GUT2P} \times k_{ab,P} \times GUT_{1,P} \cdots S9$$

$$J_{GUT1P,GUT2P} = f_{GUT1P,GUT2M} \times k_{ab,P} \times GUT_{1,P} \cdots S10$$

$$J_{GUT2P,CENP} = k_{ab,P} \times GUT_{2,P} \cdots S11$$

$$J_{CENP,CL} = \left(\frac{CL_P}{V_{1,P}}\right) \times CEN_P \cdots S12$$

$$J_{CENP,PERP} = \left(\frac{Q_P}{V_{1,P}}\right) \times CEN_P \cdots S13$$

$$J_{PERP,CENP} = \left(\frac{Q_P}{V_{2,P}}\right) \times PER_P \cdots S14$$

$$J_{GUT1M,LOST} = f_{GUT1M,LOST} \times k_{ab,M} \times GUT_{1,M} \cdots S15$$

$$J_{GUT1M,GUT2M} = f_{GUT1M,GUT2M} \times k_{ab,M} \times GUT_{1,M} \cdots\cdots S16$$

$$J_{GUT2M,CENM} = k_{ab,M} \times GUT_{2,M} \cdots\cdots S17$$

$$J_{CENM,CL} = \left(\frac{CL_M}{V_{1,M}}\right) \times CEN_M \cdots\cdots S18$$

$$J_{CENM,PERM} = \left(\frac{Q_M}{V_{1,M}}\right) \times CEN_M \cdots\cdots S19$$

$$J_{PERM,CENM} = \left(\frac{Q_M}{V_{2,M}}\right) \times PER_M \cdots\cdots S20$$

$$\frac{dGUT_{1,P}}{dt} = -J_{GUT1P,LOST} - J_{GUT1P,GUT2P} - J_{GUT1P,GUT2M} \cdots\cdots S21$$

$$\frac{dGUT_{2,P}}{dt} = J_{GUT1P,GUT2P} - J_{GUT2P,CENP} \cdots\cdots S22$$

$$\frac{dCEN_P}{dt} = J_{GUT2P,CENP} + J_{PERP,CENP} - J_{CENP,PERP} - J_{CENP,CL} \cdots\cdots S23$$

$$\frac{dPER_P}{dt} = J_{CENP,PERP} - J_{PERP,CENP} \cdots\cdots S24$$

$$\frac{dGUT_{1,M}}{dt} = -J_{GUT1M,LOST} - J_{GUT1M,GUT2M} \cdots\cdots S25$$

$$\frac{dGUT_{2,M}}{dt} = J_{GUT1P,GUT2M} + J_{GUT1M,GUT2M} - J_{GUT2M,CENM} \cdots\cdots S26$$

$$\frac{dCEN_M}{dt} = J_{GUT2M,CENM} + f_{met} \times J_{CENP,CL} + J_{PERM,CENM} - J_{CENM,PERM} - J_{CENM,CL} \cdots\cdots S27$$

$$\frac{dPER_M}{dt} = J_{CENM,PERM} - J_{PERM,CENM} \cdots\cdots S28$$

$$C_P = \frac{CEN_P}{V_{1,P}} \cdots\cdots\cdots\cdots\cdots\cdots\cdots\cdots\cdots\cdots\cdots\cdots\cdots S29$$

$$C_M = \frac{CEN_M}{V_{1,M}} \cdots\cdots\cdots\cdots\cdots\cdots\cdots\cdots\cdots\cdots\cdots\cdots\cdots S30$$

$$C_{P,u} = f_{u,p} \times C_P \cdots\cdots\cdots\cdots\cdots\cdots\cdots\cdots\cdots\cdots\cdots\cdots\cdots S31$$

$$C_{M,u} = f_{u,M} \times C_M \cdots\cdots\cdots\cdots\cdots\cdots\cdots\cdots\cdots\cdots\cdots\cdots\cdots S32$$

Sotorasib PK Model Equations:

$$\frac{A_{Lumen}}{dt} = -ka \times \ A_{Lumen} \ldots\ldots\ldots\ldots\ldots\ldots\ldots\ldots\ldots\ldots\ldots\ldots\ldots\ldots\ldots\ldots\ldots\ldots\ldots\ldots\ldots\ldots\ldots\ldots\ldots\ldots\ldots\ldots \text{S33}$$

$$\frac{Ac}{dt} = ka \times \ A_{Lumen} \times \frac{F}{100} - \left(\frac{CL}{Vc}\right) \times Ac - KT \times \left(\frac{Ac}{Vc} - \frac{\left(\frac{TUM}{V_T}\right)}{kpuu}\right) \ldots\ldots\ldots\ldots\ldots\ldots\ldots\ldots\ldots\ldots\ldots\ldots \text{S34}$$

$$\frac{TUM}{dt} = KT \times \left(\frac{Ac}{Vc} - \frac{\left(\frac{TUM}{V_T}\right)}{kpuu}\right) - K_{on} \times KRAS_F \times \frac{TUM}{V_T} + \ K_{off} \times KRAS_I \ldots\ldots\ldots\ldots\ldots\ldots\ldots \text{S35}$$
